# Immune signature of metastatic breast cancer: Identifying predictive markers of immunotherapy response

**DOI:** 10.18632/oncotarget.17653

**Published:** 2017-05-07

**Authors:** Ji-Yeon Kim, Eunjin Lee, Kyunghee Park, Woong-Yang Park, Hae Hyun Jung, Jin Seok Ahn, Young-Hyuck Im, Yeon Hee Park

**Affiliations:** ^1^ Division of Hematology-Oncology, Department of Medicine, Samsung Medical Center, Sungkyunkwan University School of Medicine, Seoul, 06351, Korea; ^2^ Samsung Genome Institute, Samsung Medical Center, Sungkyunkwan University School of Medicine, Seoul, 06351, Korea; ^3^ Biomedical Research Institute, Samsung Medical Center, Sungkyunkwan University School of Medicine, Seoul, 06351, Korea; ^4^ Samsung Advanced Institute for Health Sciences and Technology, Sungkyunkwan University School of Medicine, Seoul, 06351, Korea

**Keywords:** breast cancer, immune checkpoint, immune signature, taxane, HER2 expression

## Abstract

In breast cancer (BC), up to 10–20% patients were known to have clinical benefit with immune checkpoint inhibitors, and biomarkers are needed for optimal use of this multi-potential therapeutic strategy. Accordingly, we conducted an experiment to identify expression of genes associated with immune checkpoints that represent potential targets of cancer immunotherapy. We performed whole-transcriptome sequencing and whole-exome sequencing using 37 refractory BC specimens. In the immune pathway gene set expression analysis, we found that HER2 expression and previous taxane treatment were positively correlated with high expression of immune gene set expression (*p* = 0.070 and 0.008, respectively). The nine genes associated with immune checkpoints - PDCD1(PD-1), CD274(PD-L1), CD276(B7-H3), CTLA-4, IDO1, LAG3, VTCN1, HAVCR2, and TNFRSF4(OX40) - interacted with each other. In addition, HER2 expression also affected the expression levels of these genes (*p* = 0.044). Lastly, expression of immune checkpoint genes and tissue-infiltrating lymphocytes were positively correlated in metastatic BCs (*p* < 0.001). In conclusion, we suggest that HER2 expression and previous taxane treatment are potential surrogate markers for high expression of immune checkpoint genes and immune pathway gene sets. Further study of the BC immune signature with large-scale, translational data sets is warranted.

## INTRODUCTION

Immune check point inhibitors such as anti-programmed death-1 (PD-1) and programmed death ligand-1 (PD-L1) antibody are regarded as the “magic bullet” for refractory solid cancer [1, 2]. They inhibit the interaction between activated T cell immune check point receptors (PD-1) and tumor cell ligand (PD-L1), which facilitates tumor immune evasion [3]. Multiple phase III trials for advanced melanoma, renal cell carcinoma and non-small cell lung cancer (NSCLC) have shown better clinical outcomes for anti-PD-1 antibody than conventional treatment [4–6].

Tumor PD-L1 expression is used as a surrogate predictive marker of PD-1 treatment [1, 6]. In NSCLC, PD-L1 expression in > 50% of tumor cells is correlated with treatment efficacy. In addition, a recent study suggested that mutation profiles can be used to determine the treatment sensitivity of anti PD-1 antibody [7]. High non-synonymous mutation, neoantigen burden and DNA repair pathway mutations were correlated with good objective responses [7, 8]. However, PD-L1 expression and mutation signature have not been formally accepted as biomarkers for immunotherapy.

Immunotherapy for breast cancer (BC) has also been studied. The phase Ib KEYNOTE-012 trial with the PD-1 antibody, pembrolizumab, for heavily-treated triple-negative BC (TNBC) demonstrated an 18.5% overall response rate and three patients persisted on this treatment regime for at least 11 months [9]. In this trial, PD-L1 positivity was defined as over 1% PD-L1 expression in cancer cells or any staining in the tumor stroma and increasing expression of PD-L1 was associated with anti-PD-1 antibody response. In addition, a phase I trial of an anti-PD-L1 antibody, atezolizumab, for metastatic TNBC was performed and showed a 33% overall response [10]. A further biomarker study for atezolizumab suggested that tumor responses were correlated with high levels of PD-L1 expression in both tumor cells and tumor-infiltrating immune cells. This study also showed that T-helper type 1 gene expression and CTLA4 expression were positively correlated with response and fractalkine (CX3CL1) was negatively done [11].

With the exception of TNBC, the benefits of immunotherapy for hormone receptor-positive BC and HER2-positive BC have not been clearly defined. Advanced estrogen receptor-positive BC with PD-L1 expression achieved a 12% overall response in a phase Ib clinical trial of pembrolizumab [12]. However, only 5% of patients exhibited a response to anti-PD-L1 antibody regardless of PD-L1 expression status [13].

Here, we sought to identify the gene expression profile associated with immune check points that were potential targets of cancer immunotherapy using advanced BC specimens. Whole transcriptome sequencing analysis could provide useful clues as to which advanced BC populations would benefit from immunotherapy and could help identify predictive markers of immune check point inhibitors.

## RESULTS

### Immune gene expression and pathway analysis

**Figure 1:**
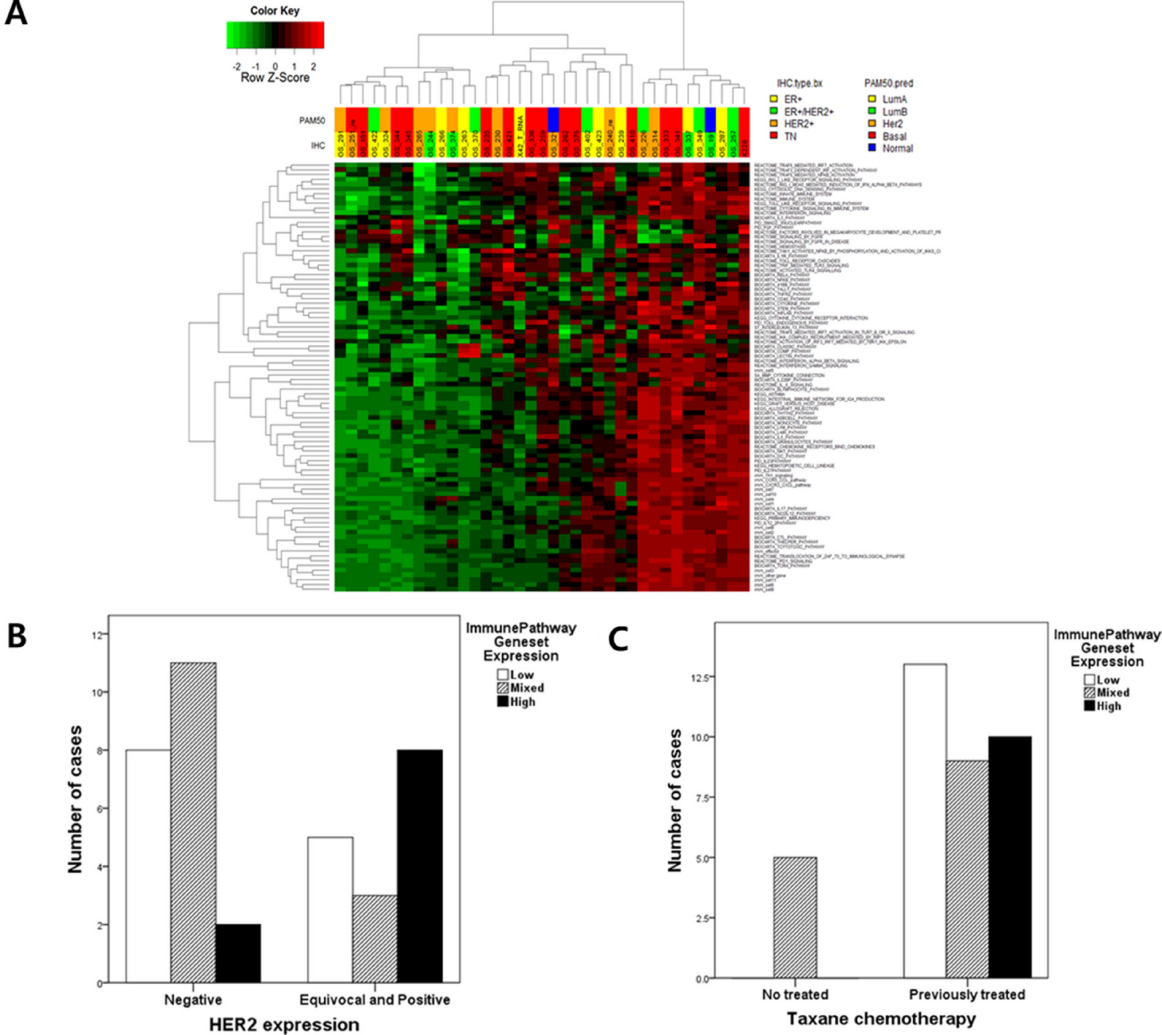
(**A**) Ninety-one immune pathway gene set enrichment analysis (GSEA) in 37 metastatic BCs : the information of 91 genesets were described in supplementary Data; (**B**) The level of immune pathway gene set expression according to HER2 expression (*p* = 0.070); According to data of GSEA of 91 immune pathway gene sets, metastatic BCs were divided into 3 groups (highly activated, mixed and inactivated immune gene sets) And then the association between the level of immune pathway gene set activation and HER2 expression were analyzed. (**C**) The level of immune pathway gene set expression according to previous taxane chemotherapy (*p* = 0.008).

Thirty-seven metastatic BC samples were analyzed in 91 gene sets associated with the immune pathway (Figure 1A). These 91 gene sets were selected as below: First, we selected 51 immune pathways, 8 pathways including IL-17 and 20 pathways including IRF7 using MSigDB gene sets. In addition, we performed literature review and selected other 16 gene sets [14]. Finally, previous our research showed that KEGG_LEISHMANIA_INFECTION gene set was probably associated to immune signature of BC and this gene set was included to analysis. After removal of duplicated gene sets, 91 gene sets were selected and analyzed (Supplementary Data).

This analysis was divided into three subgroups. One group consisting of 10 BCs showed high expression in nearly all immune pathway gene sets, while thirteen BCs showed no immune pathway activation. The third group exhibited = Of 91 gene sets, TRF3, SMAD2, TLR4, CD40 and the TOLL endogenous pathway were related to survival duration in metastatic BC. However, these gene sets did not interact with one another (Supplementary Figure 2).

Among these subgroups, we found that the HER2 immunohistochemical expression score was marginally associated with immune signature clustering (*p* = 0.070, Fischer's exact test) (Figure 1B). Other clinical factors did not affect immune pathway activation with the exception of previous taxane chemotherapy (*p* = 0.008) (Figure 1C, Table 1 and Supplementary Table 1).

**Table 1:** Impact of clinicopathological characteristics on immune signature (*N* = 37)

**(A) Immune pathway**	**Low**	**Mixed**	**High**	***p*-value**
Immunohistochemistry				
Estrogen receptor (ER)				.441
Positive	7 (18.9)	4 (10.8)	4 (10.8)	
Negative	6 (16.2)	10 (27.0)	6 (16.2)	
Progesterone receptor (PgR)				.107
Positive	3 (8.1)	3 (8.1)	6 (16.2)	
Negative	10 (27.0)	11 (29.7)	4 (10.8)	
HER2 score				.070
0	5 (13.5)	6 (16.2)	2 (5.4)	
1	3 (8.1)	5 (13.5)	0	
2	1 (2.7)	0	4 (10.8)	
3	4 (10.8)	3 (8.1)	4 (10.8)	
Ki-67				.957
Low	4 (10.8)	2 (5.4)	1 (2.7)	
High	9 (24.3)	12(32.4)	9 (24.3)	
Chemotherapeutic regimen				
Taxane				.008
Previously treated	13 (35.1)	9 (24.3)	10 (27.0)	
Untreated	0	5 (13.5)	0	
Anthracycline				.999
Previously treated	12 (35.1)	13 (35.1)	9 (21.6)	
Untreated	1 (2.7)	1 (2.7)	1 (2.7)	
**(B) Immune checkpoint gene**	**Low**	**Mixed**	**High**	***p*-value**
Immunohistochemistry				
Estrogen receptor (ER)				.999
Positive	6 (16.2)	5 (13.5)	4 (10.8)	
Negative	8 (21.6)	8 (21.6)	6 (16.2)	
Progesterone receptor (PgR)				.247
Positive	5 (13.5)	2 (5.4)	5 (13.5)	
Negative	9 (24.3)	11 (29.7)	5(13.5)	
HER2 score				.044
0	6 (16.2)	5 (13.5)	2 (5.4)	
1	3 (8.1)	5 (13.5)	0	
2	1 (2.7)	0	4 (10.8)	
3	4 (10.8)	3 (8.1)	4 (10.8)	
Ki-67				.915
Low	4 (10.8)	2 (5.4)	1 (2.7)	
High	10 (27.0)	11 (29.7)	9 (24.3)	
Chemotherapeutic regimen				
Taxane				.105
Previously treated	13 (35.1)	9 (24.3)	10 (27.0)	
Untreated	1 (2.7)	4 (10.8)	0	
Anthracycline				.999
Previously treated	13 (35.1)	12 (32.4)	9 (24.3)	
Untreated	1 (2.7)	1 (2.7)	1 (2.7)	
**(C) TIL markers**	**Low**	**Mixed**	**High**	***p*-value**
Immunohistochemistry				
Estrogen receptor (ER)				.762
Positive	2 (5.4)	5 (13.5)	7(18.9)	
Negative	6 (16.2)	8 (21.6)	9(24.3)	
Progesterone receptor (PgR)				.182
Positive	1 (2.7)	3 (8.1)	8 (21.6)	
Negative	7 (18.9)	10 (27.0)	8 (21.6)	
HER2 score				.357
0	5 (13.5)	3 (8.1)	5 (13.5)	
1	2 (5.4)	4 (10.8)	2 (5.4)	
2	0	1 (2.7)	4 (10.8)	
3	1 (2.7)	5 (13.5)	5 (13.5)	
Ki-67				.553
Low	1 (2.7)	4 (10.8)	2 (5.4)	
High	7 (18.9)	9 (24.3)	14 (37.8)	
Chemotherapeutic regimen				
Taxane				.695
Previously treated	8 (21.6)	11 (29.7)	13 (35.1)	
Untreated	0	2 (5.4)	3 (8.1)	
Anthracycline				.999
Previously treated	7 (18.9)	12 (32.4)	15 (40.5)	
Untreated	1(2.7)	1(2.7)	1(2.7)	

### Immune checkpoint gene expression

**Figure 2:**
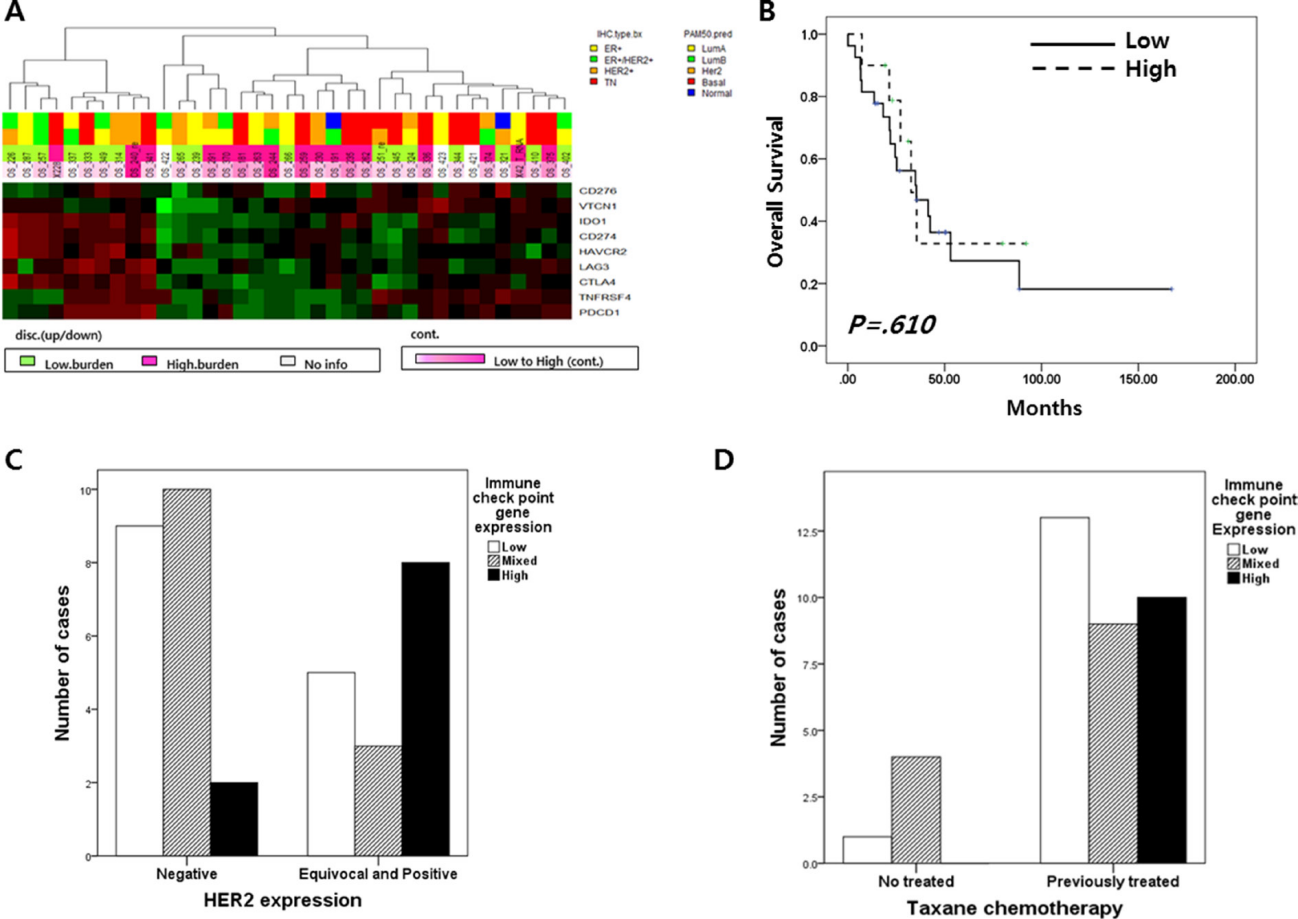
(**A**) Nine immune checkpoint gene (CD276, CD274, VTCN1, IDO1, HAVCR2, LAG, CTLA4, TNFRSF4 and PDCD1) expression profile in 37 metastatic BC; 10 breast cancer had high expression of nine immune check point genes and 27 did not. (**B**) Overall survival according to the level of immune checkpoint gene expression;(**C**) The level of immune checkpoint gene expression according to HER2 expression(*p* = 0.044); (**D**) The level of immune checkpoint gene expression according to previous taxane chemotherapy (*p* = 0.105).

Immune checkpoint gene expression, which is associated with treatment responses of immune check point inhibitors, was also analyzed. We evaluated nine genes previously reported to be targets of immune checkpoint inhibitors: PDCD1 (PD-1), CD274 (PD-L1), CD276 (B7-H3), CTLA-4, IDO1, LAG3, VTCN1, HAVCR2 and TNFRSF4 (OX40) (Figure 2A). Six of 9 genes, CD274, CTLA4, IDO1, LAG3 and HAVCR2, were similarly expressed in each metastatic BC sample. The expression patterns of TNFRSF4 and PDCD1 were also similar to one another.

Subgroups divided according to expression pattern of the 9 immune checkpoint genes did not have any distinct clinical characteristics, including survival duration (Figure 2B). However, HER2 immunohistochemical expression scores were related to immune check point gene expression (*p* = 0.044; Table 1 and Figure 2C) and previous taxane treatment was marginally affected to these gene expression ((*p* = 0.105; Table 1 and Figure 2D).

### Tumor-infiltrating lymphocytes

Tumor-infiltrating lymphocyte markers: CD3, CD4, CD8, CD20 and CD163 were evaluated using RNA-Seq data. Because CD3 was composed of CD3E, CD3G and CD3D, we analyzed seven TIL markers (CD3D, CD3E, CD3G, CD4, CD8, CD20 and CD163) [15].

**Figure 3:**
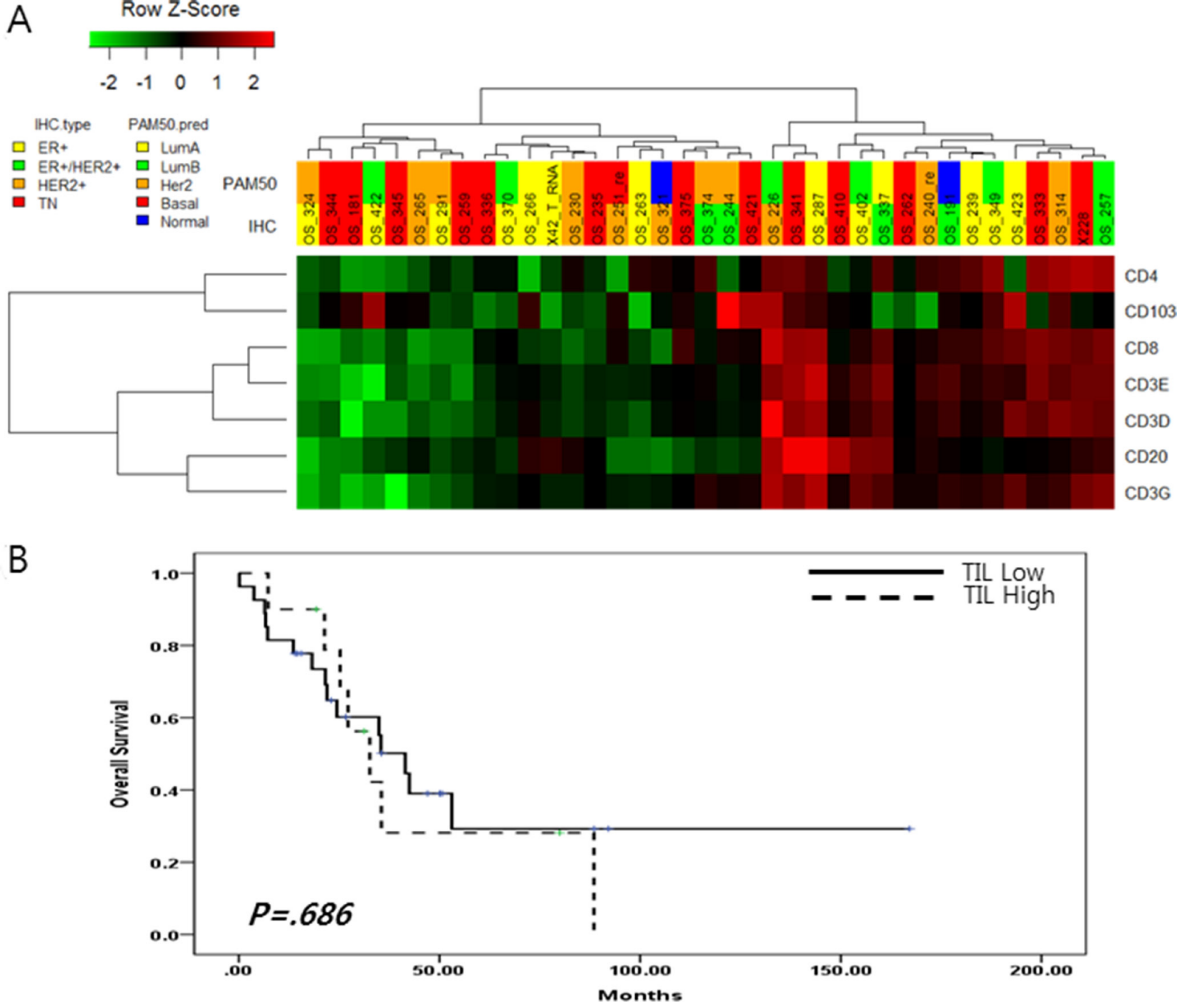
(**A**) Tumor infiltrating lymphocyte markers expression in 37 metastatic BC; (**B**) Overall survival according to the level of tumor infiltrating lymphocyte markers.

In this analysis, 37MBCs were divided into two groups according to gene expression pattern (Revised Figure 3A). One group included 16 MBCs had high expression of CD8, CD20, CD3E, CD3D and CD3G and the other did remains of MBCs. (Figure 3A). Based on this gene expression pattern, survival analysis showed that the expression of TIL markers did not influence into BC prognosis (*p* = 0.947) (Figure 3B).

Further analysis suggested that these groups were not associated with BC subtype or any clinical characteristics of metastatic BC, but no baseline characteristics were found to have a impact on lymphocyte infiltration (Table 1C).

We analyzed the relationship between the expression of nine immune check point genes and seven tumor-infiltrating lymphocyte markers. In this analysis, BC with high expression of immune check point genes also highly expressed CD8, CD20 and CD3 (*p <* 0.001) (Figure 2A and Figure 3A).

### Relationship between mutation burden and immune checkpoint gene expression

**Figure 4:**
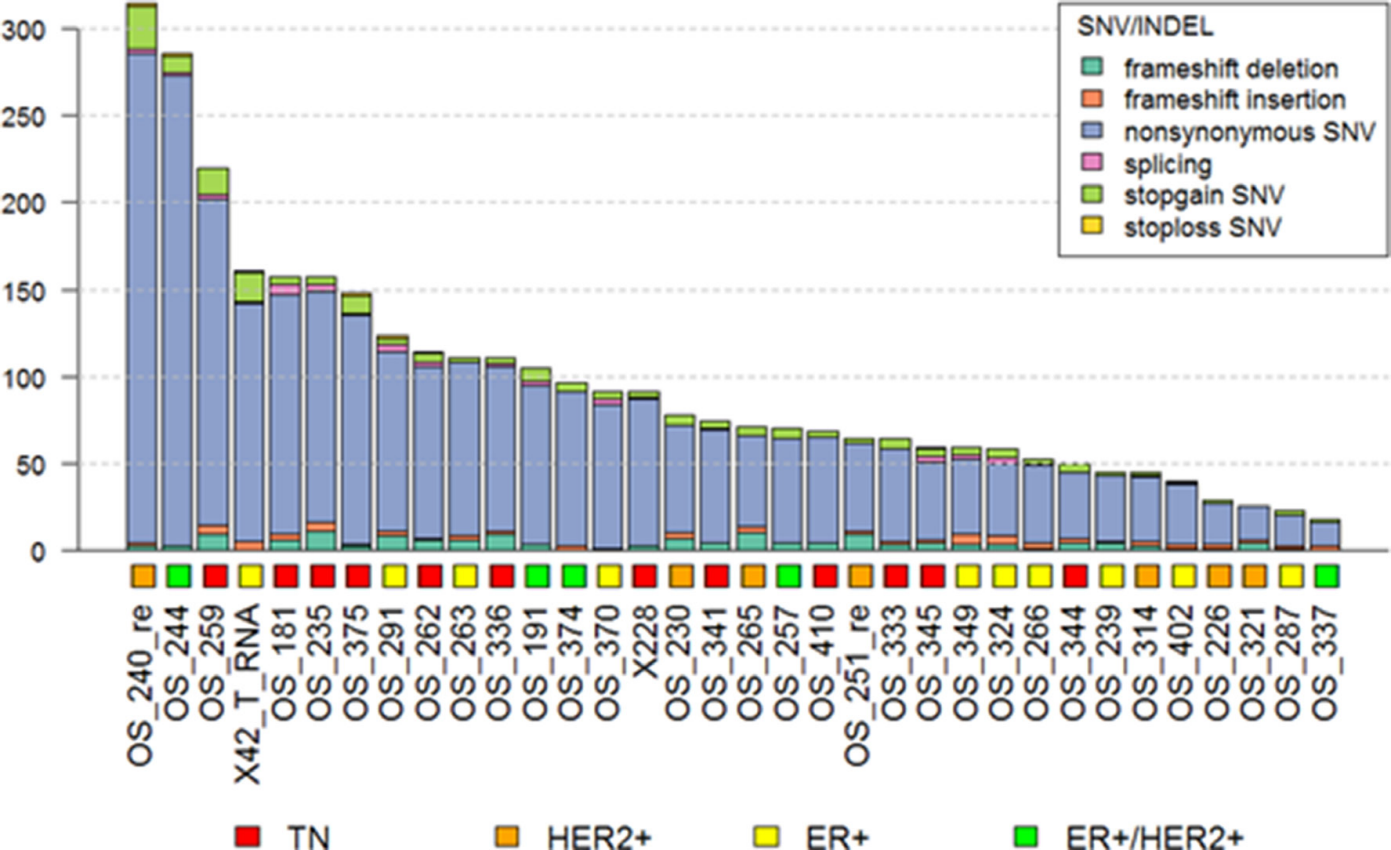
Mutation burden in metastatic BCs

Mutation burden, defined as the number of non-synonymous mutations, was checked in 34 metastatic BC samples by analyzing whole-exome sequencing data (Figure 4). The median number of non-synonymous mutations was 72.5 and this was used as the cut-off value for mutation burden (Table 2). In this analysis, non-synonymous single nucleotide variants (SNVs) were most commonly detected in metastatic BC. Low frequency frameshift deletion and stop gain SNVs were also observed.

**Table 2:** Impact of clinicopathological characteristics on mutation burden (*N* = 34)

Mutation burden	Low	High	*p-*value
Age (median) 45.1 ± 11.0, range 26.5–75.7			.807
< 40 years old	8(23.5)	6(17.6)	
≥ 40 years old	9(26.5)	11(32.4)	
Estrogen receptor (ER)			.481
Positive	9(26.5)	12(35.3)	
Negative	8(23.5)	5(14.7)	
Progesterone receptor (PgR)			.999
Positive	12(35.3)	12(35.3)	
Negative	5(14.7)	5(14.7)	
HER2 score			.379
0	5(14.7)	6(17.6)	
1	2(5.9)	5(14.7)	
2	4(11.8)	1(2.9)	
3	6(17.6)	5(14.7)	
Subtype			.404
HR*+HER2-	6(17.6)	4(11.8)	
HR+HER2+	2(5.9)	3(8.8)	
HR-HER2-	4(11.8)	8(23.5)	
HR-HER2+	5(14.7)	2(5.9)	
Intrinsic subtype			.404
Luminal A	2(5.9)	3(8.8)	
Luminal B	6(17.6)	4(11.8)	
Basal-like	4(11.8)	8(23.5)	
HER2-enriched	5(14.7)	2(5.9)	
Ki-67			.842
Low	4(10.8)	1(2.7)	
High	10(27.0)	8(21.6)	
Visceral metastasis			.999
Yes	10(29.4)	10(29.4)	
No	7(20.6)	7(20.6)	
Chemotherapeutic regimen			
Taxane			.601
Previously treated	16(47.0)	14(41.2)	
Untreated	1(2.9)	3(17.6)	
Anthracycline			.999
Previously treated	15(44.1)	14(41.2)	
Untreated	2(5.9)	1(2.9)	
Number of chemotherapy			.728
≤ 3	9(26.5)	11(32.5)	
> 3	8(23.5)	6(17.6)	

* Hormone receptor

Mutation burden was not associated with clinical characteristics such as subtype, intrinsic subtype, previous treatment history or BRCA1/2 mutation status (Table 2). Neither gene expression nor gene set pathway score was associated with mutation burden (Supplementary Figure 3). Gene expression of immune checkpoint genes did not have any association with mutation burden. Also, immune checkpoint pathway score did not interact with mutation burden (Figure 2A).

### Clinical characteristics of metastatic BC

We enrolled 54 patients with metastatic BC. RNA sequencing was performed on 37 patients and WES was on 34 patients. DNA and RNA extraction failure was the cause of RNA-Seq and WES failure.

The demographic and clinical features of 37 patients are summarized in Supplementary Table 2. The median age of the enrolled patients was 45.1 years, and 35.1% (13 patients) had TNBC. With regard to intrinsic subtype, 14 of 37 patients (37.8%) had basal-like subtype BC. Testing for BRCA1/2 mutation was performed in five patients, and a germline BRCA1 and/or BRCA2 mutation was detected in three patients. Visceral metastasis was found in 15 patients; 8 patients had brain metastasis and the others had liver metastasis. On average, patients with metastatic BC received more than three chemotherapeutic agents for palliative treatment. Thirty-six of 37 patients received anthracycline-containing cytotoxic chemotherapy and 32 were treated with taxane chemotherapy. All patients with ER-positive BC were treated with tamoxifen and/or non-steroidal aromatase inhibitor. Anti-HER2 treatment was administered to all patients with HER2-positive BC. Time to RNA-Seq from diagnosis with metastatic BC varied according to BC subtype (Supplementary Table 2). For ER-HER2-positive BC, mean time to RNA-Seq was 29.3 months (range 5.5–69.7) compared with 4.3 months (range, 0.0–36.7) for ER-HER2-negative BC.

## DISCUSSION

This study sought to identify an immune signature that would facilitate the development of therapeutic strategies for metastatic BC. We suggested that the level of HER2 expression was positively correlated to the expression level of immune check point genes in metastatic BC. Moreover, previous taxane treatment might influence immune signature. However, hormone receptor expression and intrinsic subtype did not affect the immune signature of metastatic BC.

Traditionally, tissue-infiltrating lymphocytes (TILs) have been investigated as immune markers of BC. CD8-positive cytotoxic T-lymphocytes provide useful information regarding patient survival and response to therapy and were more frequently observed in ER-negative BC [16]. In addition, CD45 and CD163, pan-leukocyte markers and M2 macrophage markers were neutral or poor prognostic markers in BC [17, 18]. In this study, the expression of CD4, CD8, PTPRC (CD45) and CD163 interacted with one another; we were able to divide groups into two categories according to whether all four genes were highly expressed. This categorization indicated that there was no difference in survival between the two groups. Moreover, clinical characteristics did not influence this categorization, while HER2 expression has a marginal impact on lymphocyte infiltration (*p* = 0.077). Accordingly, we suggested that TIL in metastatic BC might not inform.

The expression of immune check point, a potential therapeutic target of BC, was also analyzed in this study. The immune check point signaling pathway, including PD-L1, CTLA-4 and IDO1, was consistently upregulated in highly immune-activated BCs. These findings indicated that expression of new immunotherapy targets was correlated to expression of PD-L1 and CTLA-4; therefore, we suggested that BCs that showed clinical response to anti-PD-1 or PD-L1 antibody might also respond well to new immune check point inhibitors, such as TNFSF40 antibody and IDO1 inhibitor. Moreover, these results suggested that a combination of immune check point inhibitors could be an effective therapeutic strategy in metastatic BC.

Hormone receptor expression did not affect immune signature in this study. TNBC immunotherapy has been focused on in prior research, rather than other subtypes of BC. According to preliminary data from clinical trials, immune check point inhibitor is more effective in TNBC. However, up to 10% of BCs with hormone receptor expression shrank in size in response to immune checkpoint inhibitor. Therefore, other stratification factors beyond hormone receptor expression need to be explored to more accurately predict response to immunotherapy. Our study suggested that HER2 expression was related to immune signature, and thus HER2 expression might be a predictive marker for immune check point inhibitor.

Lastly, previous taxane treatment might be associated with the expression of immune check point genes. Although only 5 patients were not treated with taxane-containing regimens, all 5 of these patients did not have high immune check point gene expression. All five BCs which did not expose taxane had been treated using three therapeutic agents and under. Therefore, we performed subgroup analysis using two clinical characteristics; taxane treatment and number of previously exposed therapeutic agents. In BC treated by under three agents, taxane treatment impacted on immune check point gene expression (*p* = 0.080) and immune pathway gene expression (*p* = 0.016) (Supplementary Figure 4).

Most clinical trials on cytotoxic chemotherapy combined with immune check point inhibitor have used nab-paclitaxel. However, previous taxane chemotherapy might induce immune check point gene expression; thus, a new strategy for improving the treatment efficacy of cytotoxic chemotherapy combination for metastatic BC might be needed.

Mutation burden did not influence the immune signature of metastatic BC in this study. Melanoma [19], lung cancer [7] which were known to be correlated between mutation burden and response of immune check point inhibitor. Melanoma and lung cancer retain non-synonymous mutations the most frequently among all cancers; in contrast, BCs have only one tenth the somatic mutations of the abovementioned cancers [20]. Accordingly, mutation burden did not appear to play a significant role in the immune signature of BC, but other factors may affect immune signature and response to immunotherapy.

Tissue-infiltrating lymphocytes, immune check point gene expression were positively correlated (*p <* 0.001). BCs with high TILs had high immune check point gene expression. Therefore, TIL might be a predictive marker of immunotherapy in metastatic BC.

We sought to demonstrate the immune signature of metastatic BC in this study. The limitations of this study included a small sample size and heterogeneous sample collection. Another limitation was that all of the specimens could not be treated with immune check point inhibitor. In terms of breast cancer, the effect of immune check point inhibitor was not revealed yet. Currently, phase III clinical trials of anti-PD-L1 antibodies in breast cancer patients are ongoing [10] and these antibodies are not approved by Food and Drug Administration (FDA) for metastatic breast cancer. Therefore, we could not have any data of metastatic breast cancer patients treated with immune check point inhibitors. Therefore, our study might be the first step to understand the status of immune biomarkers in breast cancer that previously came up in other cancers in spite of some limitations.

However, translational analysis with regard to whole exome sequencing and RNA-Seq is the first step to understanding the immune signature of metastatic BC and immunotherapy response patterns in metastatic BC. Thus, this study could determine which BC types would respond to immune check point inhibitor.

In conclusion, we suggest that metastatic BC with HER2 expression and previous taxane treatment might express immune check point genes, immune pathway gene sets and tissue-infiltrating lymphocytes at high levels. Further investigation of immune signature in BC with large-scale translational studies is warranted.

## MATERIALS AND METHODS

### Patients

This study was conducted as a prospective explorative analysis of patients with metastatic BC at Samsung Medical Center. Women diagnosed with stage IV BC or recurrent BC after curative treatment on diagnostic examination and a staging work-up (breast magnetic resonance imaging [MRI], chest computed tomography [CT] scan, abdominal CT scan, bone scan, and/or positron emission tomography [PET]-CT scans if indicated) were included.

All patients provided written informed consent, and study approval was obtained from the Institutional Review Board of Samsung Medical Center, Seoul, Korea (IRB No: SMC 2012-08-065).

### Immunohistochemical (IHC) staining

Two experienced pathologists reviewed all pathology specimens to determine IHC staining for ER, PgR, and HER2. ER and PgR positivity were defined using Allred scores ranging from 3 to 8 based on IHC using antibodies to ER (Immunotech, Marseille, France) and PgR (Novocastra Laboratories Ltd., Newcastle upon Tyne, UK). HER2 status was evaluated using a specific antibody (Dako, Glostrop, Denmark) and/or silver n situ hybridization (SISH). Grades 0 and 1 for HER2, as assessed by IHC, were defined as a negative result, and grade 3 was defined as a positive result. Amplification of HER2 rated as 2+ by IHC was confirmed by SISH. Ki67 IHC analyses were performed by independent semiquantitative and quantitative methods (Dako). Triple negativity was defined as a lack of expression of ER, PgR, and HER2.

### DNA and RNA extraction

Tumors consisting of over 75% malignant cells were dissected under microscopy from 4-mm unstained sections by comparison with an H&E-stained slide, and genomic DNA was extracted using a Qiagen DNA FFPE Tissue kit (Qiagen, Hilden, Germany) according to the manufacturer's instructions. After extraction, concentration as well as 260/280 and 260/230 nm ratios were measured by spectrophotometry (ND1000, NanoDrop Technologies, ThermoFisher Scientific, MA, USA). Each sample was then quantified using a Qubit fluorometer (Life Technologies, Carlsbad, CA, USA). Genomic DNA with a total yield > 10 ng was used for library preparation.

Areas containing representative invasive breast carcinoma were outlined on the slide. Total RNA was then extracted using a High Pure RNA Paraffin kit (Roche Diagnostic, Mannheim, Germany) and the RNA concentration and 260/280- and 260/230-nm ratios were measured using a NanoDrop ND-1000 Spectrophotometer (NanoDrop Technologies, Rockland, DE, USA). Samples with less than 1 mg/μL total RNA even after concentration with a SpeedVacTM concentrator (Thermo Scientific™, Waltham, MA, USA) were excluded from downstream analysis.

### Whole-exome sequencing

Poor quality reads were filtered out and aligned to the human reference genome (hg19) using Burrows-Wheeler Alignment tool (BWA, version 0.7.5a). In order to convert Sequence Alignment and Mapping (SAM) files into Binary Alignment and Mapping files (BAM), we used SAMtools (version 0.1.19). Polymerase chain reaction (PCR) duplicates were removed from the BAM files by Picard (version 1.93, http://broadinstitute.github.io/picard/) and SAMtools before variant calling. The Genome Analysis Toolkit (GATK, version 2.4.7) was used to recalibrate base quality and optimize local realignment. Single nucleotide variants (SNVs) and indels were called using muTect (version 1.1.4) and Varscan2 (version 2.3.5) using default parameter settings. Copy number variations were detected by CONTRA (version 2.0.4). Variants were annotated using ANNOVAR, with gene, chromosomal information, exonic function function (synonymous, non-synonymous, stop gain, non-frameshift or frameshift indel), amino acid change, allele frequency in frequency in public databases such as 1000 Genomes Project (February 2012 version) and dbSNP version (version 132).

Variants chosen for further statistical analyses were located in the exonic regions with sufficient coverage (minimum depth of coverage ≥ 8) and variant allele frequency (VAF ≥ 0.1). Synonymous variants were filtered out. Read alignments were manually investigated using the Integrative Genomic Viewer (http://www.broadinstitute.org/igv/).

Fisher's exact test was used for the analysis of mutations and polymorphic variants, to discover variants that were enriched in patients with favorable outcomes. *P*-values < 0.05 were considered significantly different. All statistical analyses, plots and heatmaps were conducted using R version 3.0.2 (http://www.R-project.org/).

### RNA-Seq analysis and normalization

After trimming poor quality bases from the FASTQ files for whole-transcriptome sequencing, we aligned the reads to human reference genome hg19 with Tophat (version 2.0.6) and performed reference-guided assembly of transcripts with Cufflinks (version 2.1.1). The alignment quality was verified with SAMtools (version 0.1.19). Transcript abundance was estimated using a count-based method with a htseq-count. Gene counts were used as input for Trimmed Mean of M values (TMM) normalization i the R package edgeR [21], and normalized counts were transformed to log2-counts per million (logCPM) by applying voom from the R package limma [22] to account for higher variability at low expression levels. Genes with zero read counts across all samples were removed for more powerful statistical analysis (Supplementary Figure 1).

### Intrinsic subtyping

We performed intrinsic subtyping with log-scaled normalized expression values using the 50-gene Prediction Analysis of Microarray (PAM50) subtype predictor as described by Parker et al. [23]. The PAM50 subtype predictor classified tumors into the following groups: Luminal A, Luminal B, HER2-enriched, basal-like, and normal-like (Supplementary Figure 1).

### Gene set enrichment analysis

To examine how overall survival-associated genes share predefined gene sets representing common processes, pathways, and underlying biological themes, we investigated sub-collections in the Molecular Signatures Database (MSigDB, version 5.0) with overall survival-associated genes using the Gene Set Enrichment Analysis (GSEA) website. We also calculated Gene Set Enrichment (GSE) scores for canonical pathways in MSigDB and several AR-related gene sets from the literature [24, 25] using the R package Gene Set Variation Analysis (GSVA). GSVA is a nonparametric method that provides sample-wise gene set enrichment scores to identify differential gene set activity. A two-sample *t-test* was then performed, and gene sets with a false discovery rate less than 0.05 were considered to show significantly different activity between the two groups. All normalization, statistical analyses, and visualization were conducted within the R statistical system (version 3.0.2).

### Survival analysis

We evaluated the association between gene expression and overall survival using the R package. Overall survival was defined as the elapsed time between the date of stage IV BC diagnosis and the date of death. For each gene, patients were grouped based on the normalized expression value of the gene, with the top 50% and the bottom 50% representing high and low expression groups, respectively. Survival curves for the two groups were estimated with the Kaplan-Meier method, and the log-rank test was used to compare overall survival curves between the two groups (*p <* 0.05). Fisher's exact test was used to identify the pathways with significant enrichment of associated genes in terms of overall survival (*p <* 0.05).

## SUPPLEMENTARY DATA FIGURES AND TABLES








