# Supplementary material for: Immune signature of metastatic breast cancer: Identifying predictive markers of immunotherapy response
Source: Oncotarget. 2017 May 7;8(29):47400–11. doi: 10.18632/oncotarget.17653 (PMC5564574; doi:10.18632/oncotarget.17653)
Supplement: Supplementary file 1 [file oncotarget-08-47400-s001.pdf]

Immune signature of metastatic breast cancer: Identifying predictive markers of immunotherapy response

Supplementary Materials

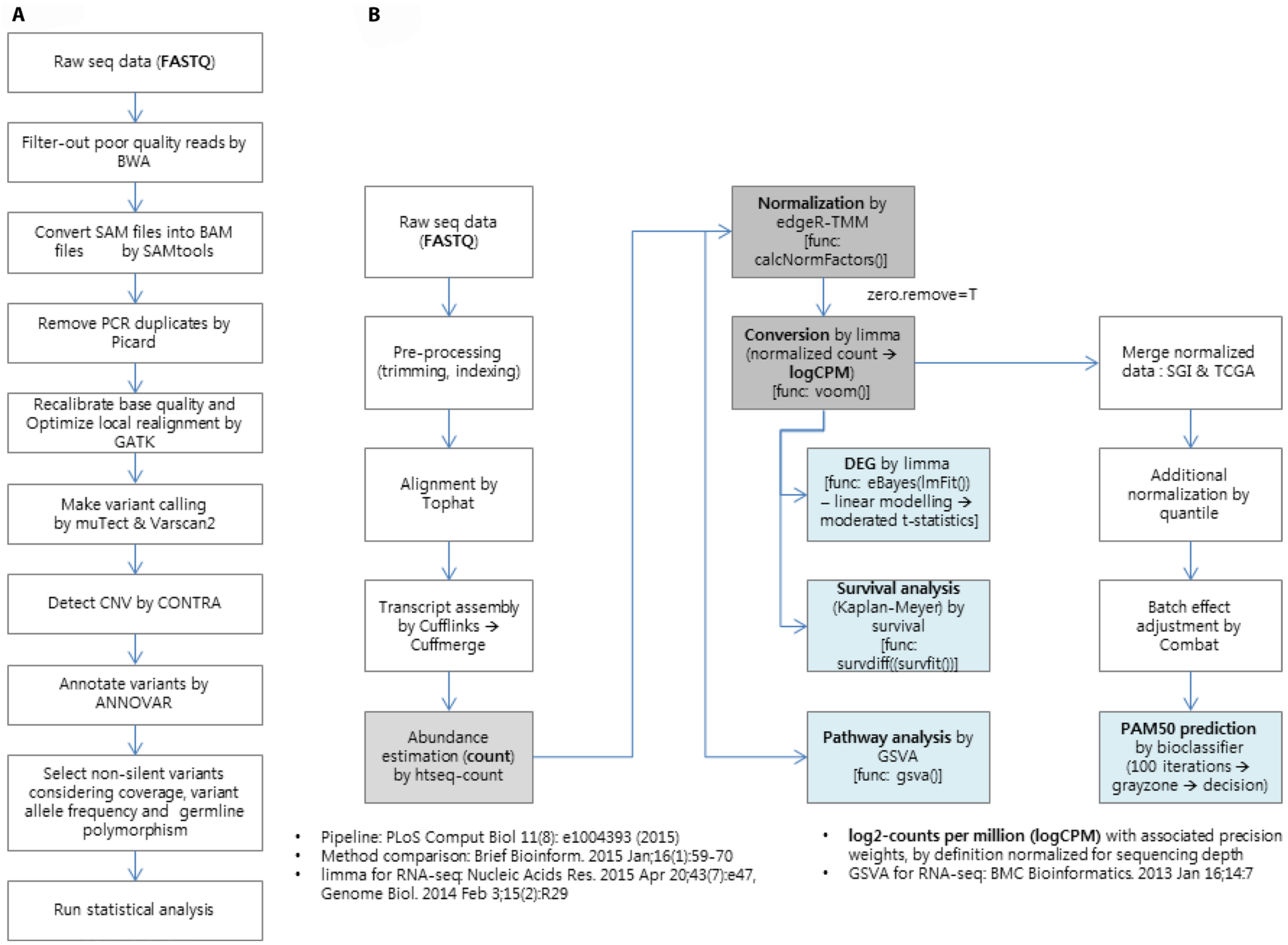

Supplementary Figure 1: Pipeline of next generation sequencing data analysis (**A**) whole exome sequencing (**B**) RNA-Seq.

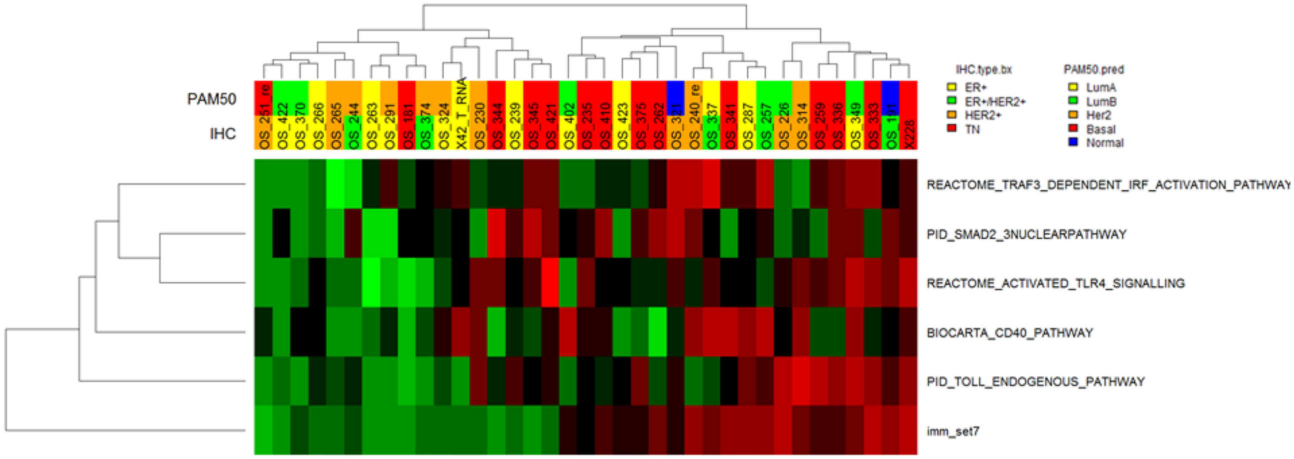

Supplementary Figure 2: Immune pathway gene set expression that associated survival duration in metastatic BC.

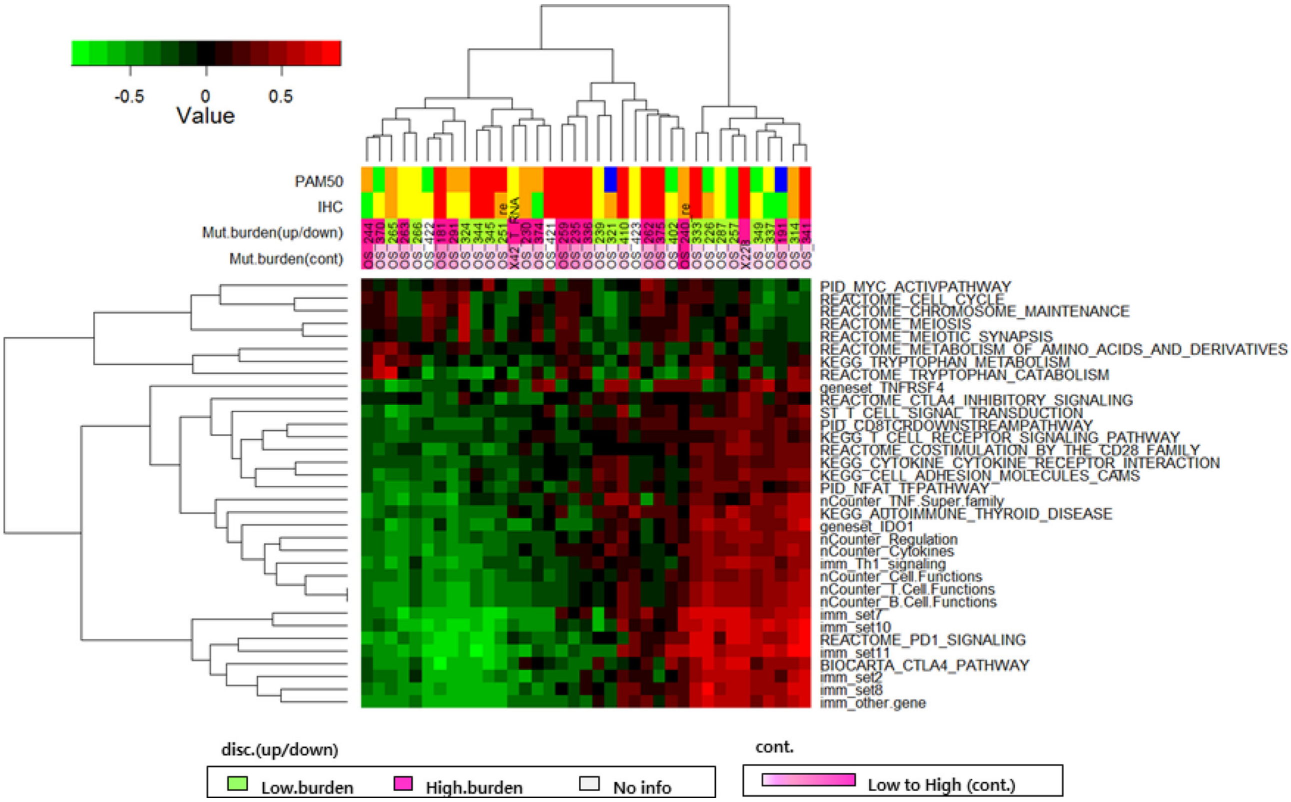

Supplementary Figure 3: The association between mutation burden and immune gene set expression.

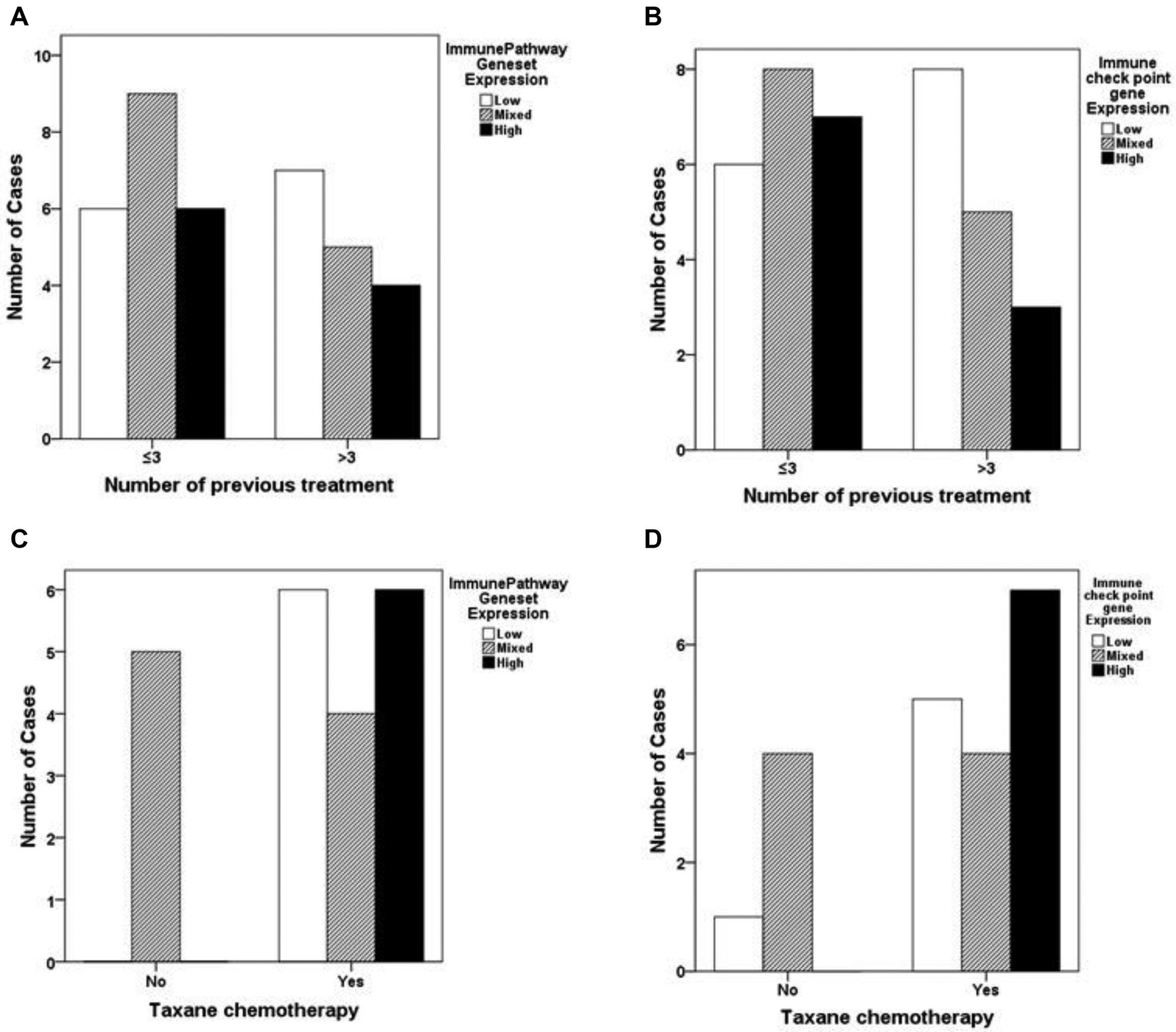

Supplementary Figure 4: The association between (**A**) immune pathway gene set expression and previous treatment; (**B**) immune check point gene expression and previous treatment; (**C**) immune pathway gene set expression and previous taxane treatment; (**D**) immune check point gene expression and previous taxane treatment.

Supplementary Table 1: Impact of clinicopathological characteristics on immune signature (*N* = 37). See Supplementary_Table_1

Supplementary Table 2: Clinicopathological characteristics of metastatic breast cancer *N* = 37. See Supplementary_Table_2

Supplementary Data 1: list_immune_pathway. See Supplementary_Data_1
