# Supplementary material for: Immune signature of metastatic breast cancer: Identifying predictive markers of immunotherapy response
Source: Oncotarget. 2017 May 7;8(29):47400–11. doi: 10.18632/oncotarget.17653 (PMC5564574; doi:10.18632/oncotarget.17653)
Supplement: Supplementary file 3 [file oncotarget-08-47400-s003.docx]

**Supplementary Table 1. Impact of clinicopathological characteristics on immune signature (N=37)**

| **(A) Immune pathway** | **Low** | **Mixed** | **High** | ***p-*value** |
| --- | --- | --- | --- | --- |
| Age (median) 45.1±11.0, range 26.5-75.7 .531 | | | | |
| <40 years old | 6 (16.2) | 4 (10.8) | 5 (13.5) |  |
| ≥40 years old | 7 (18.9) | 10 (27.0) | 5 (13.5) |  |
| Subtype |  |  |  | .349 |
| HR^*^+HER2- | 6 (16.2) | 4 (10.8) | 2 (5.4) |  |
| HR+HER2+ | 2 (5.4) | 0 | 3 (8.1) |  |
| HR-HER2- | 3 (8.1) | 7 (18.9) | 3 (8.1) |  |
| HR-HER2+ | 2 (5.4) | 3 (8.1) | 2 (5.4) |  |
| Intrinsic subtype |  |  |  | .608 |
| Luminal A | 2 (5.4) | 3 (8.1) | 2 (5.4) |  |
| Luminal B | 2 (5.4) | 1 (2.7) | 3 (8.1) |  |
| Basal-like | 4 (10.8) | 7 (18.9) | 3 (8.1) |  |
| Normal-like | 0 | 1 (2.7) | 1 (2.7) |  |
| HER2-enriched | 5 (13.5) | 2 (5.4) | 1 (2.7) |  |
| Visceral metastasis |  |  |  | .371 |
| Yes | 5 (13.5) | 4 (10.8) | 6 (16.2) |  |
| No | 8 (21.6) | 10 (27.0) | 4 (10.8) |  |
| TP53 mutation |  |  |  | .834 |
| Wild type | 7 (18.9) | 8 (21.6) | 5 (13.5) |  |
| Mutation | 5 (13.5) | 4 (10.8) | 5 (13.5) |  |
| Unknown | 1 (2.7) | 2 (5.4) | 0 |  |
| PIK3CA mutation |  |  |  | .914 |
| Wild type | 9 (24.3) | 8 (21.6) | 7 (18.9) |  |
| Mutation | 3 (8.1) | 4 (10.8) | 3 (8.1) |  |
| Unknown | 1 (2.7) | 2 (5.4) | 0 |  |
| Number of chemotherapy |  |  |  | .701 |
| ≤3 | 6 (16.2) | 9 (24.3) | 6 (16.2) |  |
| >3 | 7 (18.9) | 5 (13.5) | 4 (10.8) |  |
| **(B) Immune checkpoint gene** | **Low** | **Mixed** | **High** | ***p-*value** |
| Age (median) 45.1±11.0, range 26.5-75.7 | |  |  | .836 |
| <40 years old | 5 (13.5) | 5 (13.5) | 5 (13.5) |  |
| ≥40 years old | 9 (24.3) | 8 (20.6) | 5 (13.5) |  |
| Subtype |  |  |  | .831 |
| HR+HER2- | 6 (16.2) | 4 (10.8) | 2 (5.4) |  |
| HR+HER2+ | 2 (5.4) | 1 (2.7) | 2 (5.4) |  |
| HR-HER2- | 4 (10.8) | 6 (16.2) | 3 (8.1) |  |
| HR-HER2+ | 2 (5.4) | 2 (5.4) | 3 (8.1) |  |
| Intrinsic subtype |  |  |  | .859 |
| Luminal A | 3 (8.1) | 2 (5.4) | 2 (5.4) |  |
| Luminal B | 2 (5.4) | 1 (2.7) | 3 (8.1) |  |
| Basal-like | 4 (10.8) | 7 (18.9) | 3 (8.1) |  |
| Normal-like | 1 (2.7) | 1 (2.7) | 0 |  |
| HER2-enriched | 4 (10.8) | 2 (5.4) | 2 (5.4) |  |
| Visceral metastasis |  |  |  | .701 |
| Yes | 6 (16.2) | 4 (10.8) | 5 (13.5) |  |
| No | 8 (21.6) | 9 (24.3) | 5 (13.5) |  |
| TP53 mutation |  |  |  | .248 |
| Wild type | 9 (24.3) | 7 (18.9) | 4 (10.8) |  |
| Mutation | 4 (10.8) | 4 (10.8) | 6 (16.2) |  |
| Unknown | 1 (2.7) | 2 (5.4) | 0 |  |
| PIK3CA mutation |  |  |  | .620 |
| Wild type | 9 (24.3) | 9 (24.3) | 6 (16.2) |  |
| Mutation | 4 (10.8) | 2 (5.4) | 4 (10.8) |  |
| Unknown | 1 (2.7) | 2 (5.4) | 0 |  |
| Number of chemotherapy |  |  |  | .452 |
| ≤3 | 6 (16.2) | 8 (21.6) | 7 (18.9) |  |
| >3 | 8 (21.6) | 5 (13.5) | 3 (8.1) |  |
| **(C) TIL markers** | **Low** | **Mixed** | **High** | ***p-*value** |
| Age (median) 45.1±11.0, range 26.5-75.7 | |  |  | .753 |
| <40 years old | 4 (10.8) | 4 (10.8) | 7 (18.9) |  |
| ≥40 years old | 4 (10.8) | 9 (24.3) | 9 (24.3) |  |
| Subtype |  |  |  | .931 |
| HR+HER2- | 3 (8.1) | 4 (10.8) | 5 (13.5) |  |
| HR+HER2+ | 0 | 2 (5.4) | 3 (8.1) |  |
| HR-HER2- | 4 (10.8) | 4 (10.8) | 5 (13.5) |  |
| HR-HER2+ | 1 (2.7) | 3 (8.1) | 3 (8.1) |  |
| Intrinsic subtype |  |  |  | .946 |
| Luminal A | 1 (2.7) | 3 (8.1) | 2 (5.4) |  |
| Luminal B | 2 (5.4) | 3 (8.1) | 6 (16.2) |  |
| Basal-like | 4 (10.8) | 4 (10.8) | 5 (13.5) |  |
| HER2-enriched | 1 (2.7) | 3 (8.1) | 3 (8.1) |  |
| Visceral metastasis |  |  |  | .999 |
| Yes | 3 (8.1) | 5 (13.5) | 7 (18.9) |  |
| No | 5 (13.5) | 8 (21.6) | 9 (24.3) |  |
| TP53 mutation |  |  |  | .020 |
| Wild type | 2 (5.4) | 11 (29.7) | 7 (18.9) |  |
| Mutation | 5 (13.5) | 1 (2.7) | 8 (21.6) |  |
| Unknown | 1 (2.7) | 1 (2.7) | 1 (2.7) |  |
| PIK3CA mutation |  |  |  | .878 |
| Wild type | 6 (16.2) | 8 (21.6) | 10 (27.0) |  |
| Mutation | 1 (2.7) | 4 (10.8) | 5 (13.5) |  |
| Unknown | 1 (2.7) | 1 (2.7) | 1 (2.7) |  |
| Number of chemotherapy |  |  |  | .835 |
| ≤3 | 4 (10.8) | 7 (18.9) | 10 (27.0) |  |
| >3 | 4 (10.8) | 6 (16.2) | 6 (16.2) |  |

* Hormone receptor
