# Supplementary material for: Immune signature of metastatic breast cancer: Identifying predictive markers of immunotherapy response
Source: Oncotarget. 2017 May 7;8(29):47400–11. doi: 10.18632/oncotarget.17653 (PMC5564574; doi:10.18632/oncotarget.17653)
Supplement: Supplementary file 4 [file oncotarget-08-47400-s004.docx]

**Supplementary Table 2. Clinicopathological characteristics of metastatic breast cancer (N=37)**

| **Characteristics** | ***N* = 37(%)** |
| --- | --- |
| Age (median) | 45.1±11.0 |
| Range | 26.5-75.7 |
| <40 years old | 15 (40.5) |
| ≥40 years old | 22 (59.5) |
| Histology |  |
| Invasive ductal carcinoma | 34 (91.9) |
| Other | 3 (8.1) |
| Subtype |  |
| HR^*^+HER2- | 12 (32.4) |
| HR+HER2+ | 5 (13.5) |
| HR-HER2- | 13 (35.1) |
| HR-HER2+ | 7 (18.9) |
| Intrinsic subtype |  |
| Luminal A | 7 (18.9) |
| Luminal B | 6 (16.2) |
| Basal-like | 14 (37.8) |
| Normal-like | 2 (5.4) |
| HER2-enriched | 8 (21.6) |
| BRCA1/2 |  |
| Wild type | 2 (5.4) |
| Mutated | 3 (8.1) |
| Not tested | 32 (86.5) |
| Cancer status |  |
| Recurred | 27 (73.0) |
| Initially metastatic | 10 (27.0) |
| Visceral metastasis |  |
| Yes | 15 (40.5) |
| Liver metastasis | 7 (18.9) |
| Brain metastasis | 8 (21.6) |
| No | 22 (59.5) |
| Biopsy site |  |
| Breast | 12 (32.4) |
| Lymph node | 7 (18.9) |
| Pleura | 7 (18.9) |
| Liver | 3 (8.1) |
| Lung | 2 (5.4) |
| Other | 6 (16.2) |
| Chemotherapy agents (average 3.24) |  |
| 1 | 8 (21.6) |
| 2 | 11 (29.7) |
| 3 | 4 (10.8) |
| ≥4 | 14 (37.8) |
| Chemotherapeutic regimen |  |
| Anthracycline | 36 (97.3) |
| Taxane | 31 (83.8) |
| Both anthracycline and taxane | 27 (73.0) |
| Hormone therapy (N=17) |  |
| Yes | 17 (100.0) |
| No | 0 (0.0) |
| HER2 targeted therapy (N=12) |  |
| Yes | 12 (100.0) |
| No | 0 (0.0) |

* Hormone receptor
